# Next-Generation Self-Powered Photodetectors using 2D Bismuth Oxide Selenide Crystals

**DOI:** 10.1021/acsanm.4c03594

**Published:** 2024-10-23

**Authors:** Pradip Kumar Roy, Kseniia Mosina, Sofia Hengtakaeh, Kalyan Jyoti Sarkar, Vlastimil Mazánek, Jan Luxa, Zdenek Sofer

**Affiliations:** Department of Inorganic Chemistry, University of Chemistry and Technology Prague, Technická 5, 166 28 Prague, Czech Republic

**Keywords:** 2D Bismuth oxide selenide, photoelectrochemical (PEC) photodetector, self-powered, broadband, band bending

## Abstract

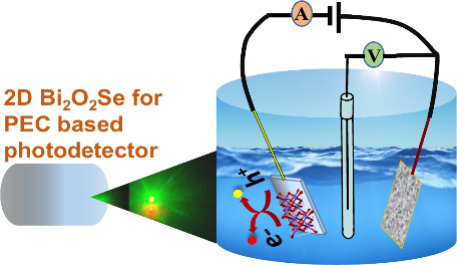

The concept of self-powered photodetectors has attracted significant attention due to their versatile applications in areas such as intelligent systems and hazardous substance detection. Among these, *p–n* junction and Schottky junction photodetectors are the most widely studied types; however, their fabrication processes are often complex and costly. To overcome these challenges, we focused on the emerging self-powered, ultrasensitive photodetector platform based on photoelectrochemical (PEC) principles. This platform leverages the unique properties of the emerging material bismuth oxide selenide (Bi_2_O_2_Se), which features a wide bandgap (∼2 eV) and a high absorption coefficient. We utilized chemical exfoliation to obtain thin layers of Bi_2_O_2_Se, enabling highly efficient photodetection. The device characterization demonstrated impressive performance metrics, including a responsivity of 97.1 μA W^–1^ and a specific detectivity of 2 × 10^8^ cm Hz ^1/2^ W^–1^. The PEC photodetector also exhibits broad-spectrum sensitivity, from blue to infrared wavelengths, and features an ultrafast response time of ∼82 ms and a recovery time of ∼86 ms, highlighting its practical potential. Moreover, these self-powered photodetectors show excellent stability in electrochemical environments, positioning them promising candidates for integration into future high-efficiency devices.

## 1 Introduction

Photodetectors play an essential role in our contemporary lives, encompassing a diverse array of applications such as security, sensing, defense, and food safety.^1^ As a result, the exploration of new photodetector devices bears significant importance for the advancement of future electronics. In this context, two-dimensional (2D) materials stand out as the pioneers due to their exceptional attributes, including an expansive surface-to-volume ratio, an adjustable band gap, and exceptional electronic properties.^2,3^ Over the past decade, there has been a remarkable surge in the study of 2D materials. Standout examples, such as graphene,^2,3^ transition-metal dichalcogenides (TMDs),^4^ and black phosphorus^5^ have garnered immense interest for their unique properties and potential applications. However, challenges such as zero band gap, susceptibility to air-induced instability, and limited electron mobility have impeded their widespread adoption. As a result, researchers continue to explore new materials that can meet the demands of next-generation electronics.

Recently, there has been successful synthesis of the emerging compound Bismuth oxide selenide (Bi_2_O_2_Se), characterized with weak interlayer electrostatic interactions.^6^ This achievement has sparked significant interest among researchers due to its moderate band gap, high mobility (1.9 K, 29,000 cm^2^ V^–1^ s^–1^), and remarkable environmental stability. These properties make Bi_2_O_2_Se a highly promising candidate for applications in optoelectronics.^6−8^ Furthermore, Bi_2_O_2_Se distinguishes itself as a superior semiconductor, with an exceptional current on/off ratio exceeding 10^6^ at room temperature.^6^ Despite being in the early stages of research, the reported optoelectronic performance results are highly encouraging.^9−19^ For instance, a successfully synthesized a Bi_2_O_2_Se/Graphene composite was employed to construct a photodetector that demonstrated remarkable photoresponse performance, even at 0 V bias, along with remarkable environmental stability.^19^ Similarly, another study reported the growth of atomically thin Bi_2_O_2_Se nanoribbons, which exhibited exceptional electron mobility (262 cm^2^ V^–1^ s^–1^), an on/off ratio surpassing 10^7^, and an impressive photoresponsivity (9.2 × 10^6^ AW^–1^).^9^ Additionally, an infrared photodetector based on 2D Bi_2_O_2_Se, showcasing both high sensitivity (65 AW^–1^) at 1200 nm and ultrafast photoresponse (∼1 ps) at room temperature.^10^ While limited advancements have been reported in this field, the current progress remains at a nascent stage, primarily focused on directly grown photodetectors with intricate device fabrication techniques.

Recently, the spotlight has shifted toward self-powered photodetectors, capturing the interest of researchers due to their capacity to function over extended periods without external energy sources. Prominent categories of self-powered photodetectors include *p–n* junction and Schottky junction photodetectors.^20,21^ The *p–n* junction employs a blend of *p*-type and *n*-type materials in its construction, while the Schottky junction replaces the *p*-type component with a metal. However, both these require intricate device fabrication procedures and can be economically burdensome. In response, we are exploring an alternative: photoelectrochemical^22−25^ based self-powered photodetectors. These self-powered photodetectors offer an array of benefits surpassing those of traditional electrochemical sensors. Beyond eliminating the need for complex device fabrication, they offer several unique advantages. Notably, their ability to operate in differential mode significantly reduces background noise and minimizes the frequency of recalibration.^26−28^ Additionally, the versatility of photoelectrochemical systems makes them suitable for a wide range of applications, such as solar cells, sensing, and imaging. Therefore, there is a compelling rationale to focus on the development of self-powered Bi_2_O_2_Se-based photodetectors that can be facilely fabricated, owing to their pronounced potential and relevance.

This study showcases a highly effective exfoliation technique tailored for Bi_2_O_2_Se, a layered material. The method, based on electrochemical processes, successfully yields of Bi_2_O_2_Se with only a few layers. Comprehensive characterization of the exfoliated samples is conducted using SEM, Raman spectroscopy, XRD, and X-ray photoelectron spectroscopy. Notably, we are the first to utilize exfoliated Bi_2_O_2_Se in a self-powered photodetector, leveraging the principles of photoelectrochemical (PEC) technology.

## 2 Experimental Section

### 2.1 Synthesis

The Bi_2_Se_2_O was prepared by direct reaction of elements in quartz ampule using Bi granules (99.9999%, 2–6 mm, Wuhan Xinrong New Materials Co.), selenium granules (99.9999%, 2–4 mm, Wuhan Xinrong New Materials Co.) and bismuth oxide (99.999%, powder, Sigma Aldrich, Czech Republic). The stoichiometric mixture corresponding to 25 g of Bi_2_O_2_Se was placed in alumina crucible inside quartz glass ampule (40 × 120 mm) and melt sealed under high vacuum (1 × 10^–3^ Pa) using oxygen-hydrogen welding torch. The ampule was placed in a muffle furnace, and the reaction mixture was heated to 960 °C at a heating rate of 1 °C/min. After maintaining this temperature for 6 h, the ampule was cooled to 920 °C at a rate of 3 °C/h, followed by a gradual cooling to room temperature at 1 °C/min. The resulting Bi_2_O_2_Se crystals were mechanically extracted from the ampule and stored in an argon-filled glovebox.

### 2.2 Instruments

Scanning Electron Microscope (SEM) was employed to explore the surface morphology of the exfoliated materials. For this analysis, we utilized a Tescan Lyra dual beam microscope equipped with an FEG electron source, applying an electron beam with an effective voltage of approximately 15 kV.

X-ray diffraction (XRD) analyses were conducted employing a Bruker D8 Discoverer powder diffractometer from Bruker in Germany. The instrument was equipped with a Cu Kα radiation source (λ = 0.15418 nm, *U* = 40 kV, *I* = 40 mA). The XRD measurements covered an angular range of 5–90° (2θ) with a step size of 0.01517° (2θ). Subsequently, the acquired XRD data set underwent analysis utilizing the High Score Plus software.

We conducted Raman spectroscopy utilizing a Renishaw Raman microscope coupled with a CCD detector. The excitation source was a green DPSS laser (532 nm, 50 mW), and the instrument’s calibration involved the standard silicon peak (520 cm^–1^) with a remarkable resolution below 1 cm^–1^. Our experimental setup employed the microscope’s 50× objective, utilizing a laser power of 0.5 mW, and an exposure duration of 25 s.

The XPS spectra were examined utilizing a SPECS system, incorporating a monochromatic X-ray source (XR 50 MF) at an energy of 1486.7 eV and a Phoibos 150 hemispherical analyzer equipped with a 2D CCD detector. The measurements were conducted under ultralow vacuum conditions, reaching pressures of 5 × 10^–10^ mbar or lower. A high-resolution XPS analysis of the exfoliated sample was performed using an ESCA Probe spectrometer (Omicron Nanotechnology Ltd., Germany). A detailed scan of the targeted core-level lines was carried out within a 20 eV energy window, while broader scans were conducted within a 40 eV energy window. The sample was positioned on a highly conductive stage made of high purity gold. Throughout the measurement process, the charging effect was mitigated using an electron gun operating at a range of 1–5 V.

All electrochemical characterizations and photosensor investigations were conducted using an Autolab PGSTAT 204 (Metrohm, Switzerland). Photoelectrochemical (PEC) photodetection employed a three-electrode configuration with the following deposited materials: working electrode (WE), platinum counter electrode, and saturated calomel reference electrode (SCE).

TEM, including high-resolution TEM and SAED, was recorded on an EFTEM Jeol 2200 FS microscope, Japan. Energy-dispersive X-ray (EDX) analysis was also conducted using a detector from Oxford Instruments in the same instrument.

The experiment employed the SZ-05-H3 LED module for illumination, producing 144 lm at a current of 500 mA, with the potential to reach an impressive 244 lm at its maximum current of 1000 mA. To ensure stable power output, a robust cooling system was utilized throughout the process. The light output was generated by four closely arranged LUXEON Z-LEDs (model LXZ1-PB01) connected in series, expertly soldered onto a thermally efficient aluminum base measuring 20 mm in width and 1.6 mm in thickness.

## 3 Results and Discussion

**Figure 1:**
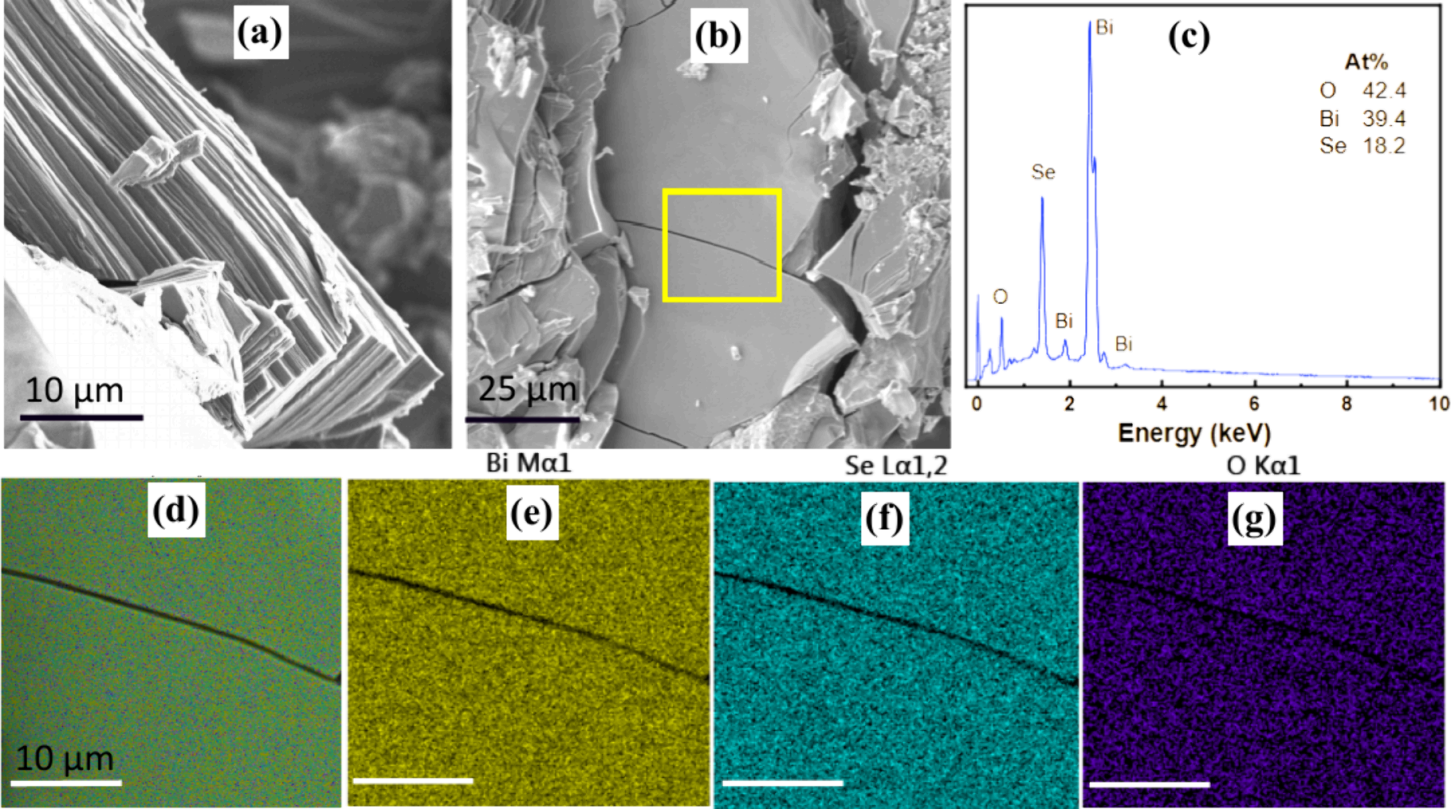
(a) Top-view SEM images of bulk Bi_2_O_2_Se displaying its layered structure. (b) Top-view images with a highlighted yellow rectangle, used for further analysis. (c) SEM EDX measurements were conducted for elemental composition analysis. (d–g) These images show the uniform distribution of the elements Bi, Se, and O throughout the bulk material. Scale used in (d–g) is 10 μm.

Bulk Bi_2_O_2_Se crystals were synthesized through flux growth in quartz ampoule. The SEM image (Figure 1a) clearly reveals the layered structure of the resultant Bi_2_O_2_Se crystal. To confirm the uniform distribution of the constituent elements (Bi, O, and Se) within the marked region (highlighted by a yellow rectangle), EDX mapping and the corresponding EDX spectrum were performed. The observed element distribution closely aligns with the expected stoichiometric ratio of approximately 2:2:1, as depicted in Figure 1b–g.

The process of electrochemical exfoliation was employed to transform bulk Bi_2_O_2_Se crystals into few-layered Bi_2_O_2_Se materials, suitable for various optoelectronic applications. In this method, the bulk crystals were mounted within a platinum holder and connected as the working electrode. Accompanying this, a platinum plate (approximately 1 × 2 cm^2^) was employed as the counter electrode (CE), while Ag/Ag^+^ (consisting of 0.01 M AgNO_3_ in acetonitrile) was adopted as the reference electrode. The electrolyte solution utilized in this process was a 0.1 M LiPF_6_ dissolved in DMF. To ensure an oxygen-free environment, the electrochemical cell was continuously purged with argon. The exfoliation process unfolded in two distinctive stages, each regulated by different potentials. Figure S1 shows the *I*–*V* curve, measured via linear sweep voltammogram (LSV) with a scanning rate of 50 mV s^–1^. Initially, a potential of −1 V was applied to the cathode to pretreat the bulk material. This was followed by applying a potential of −2 V to the working electrode for a duration of 2 min, promoting the preliminary ion intercalation. Subsequently, Li^+^ ions infiltrated the crystal interlayers, consequently inducing the exfoliation of crystals when the potential reached −5 V. The entire exfoliation process lasted for 4 h, during which the crystals underwent expansion and noticeable separation. Following the completion of the exfoliation procedure, all resultant material was collected from the electrochemical cell, and subjected to centrifugation in acetonitrile to eliminate any residual unexfoliated crystals and traces of LiPF_6_. The material was then resuspended in DMF for further use.

**Figure 2:**
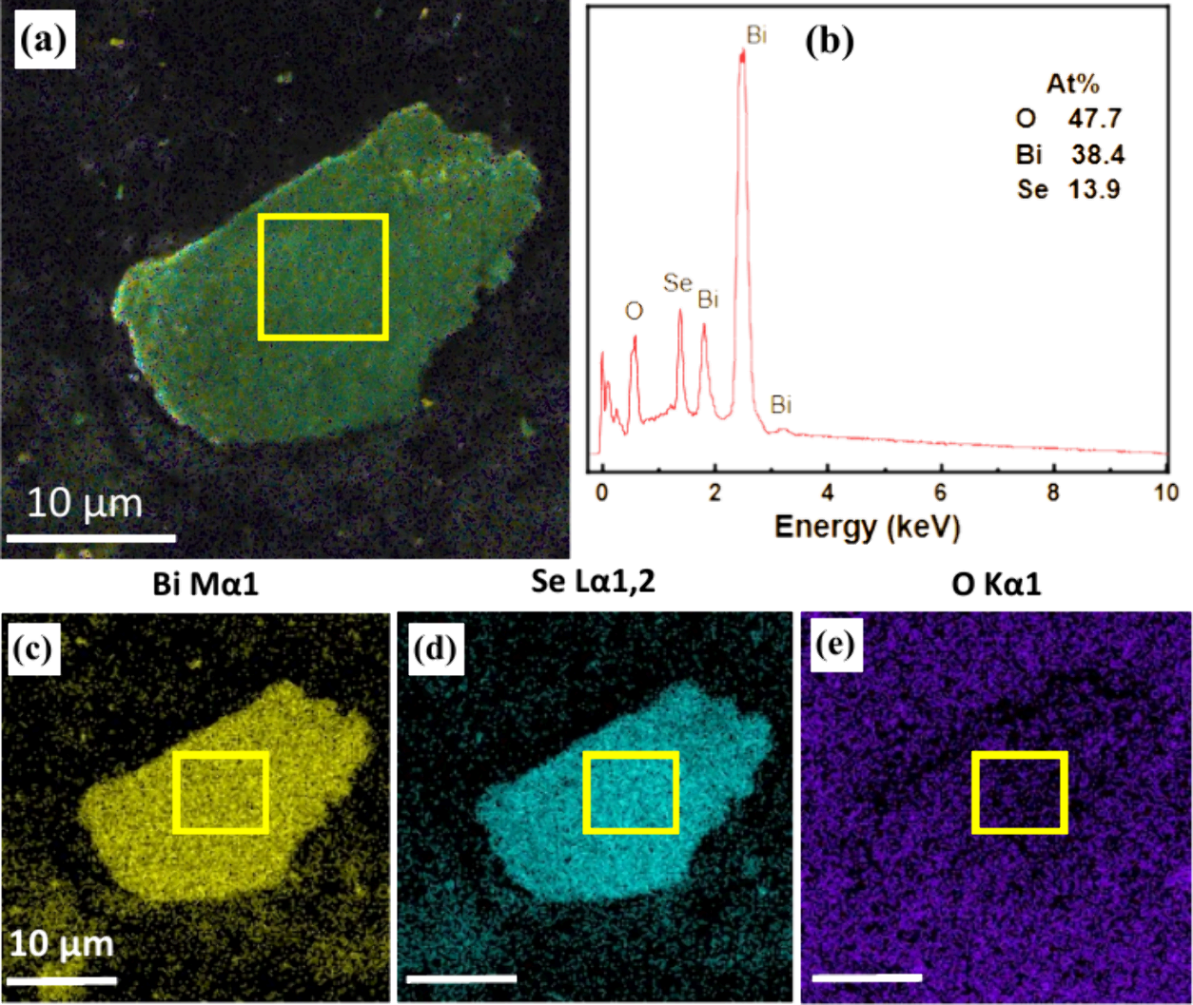
Characterization of Layered Exfoliated Bi_2_O_2_Se (a) Top-View SEM Image of Layered Exfoliated Bi_2_O_2_Se, with a highlighted yellow rectangle indicating the region selected for further analysis. (b) SEM EDX measurements with elemental composition analysis. (c–e) These images illustrate the consistent distribution of the elements Bi, Se, and O_2_ within the bulk material. Scale of 10 μm is employed in (c–e) for size calibration.

Upon initial characterization of the exfoliated material, SEM was employed. This analysis revealed a distinct flake-like structure, as visually depicted in Figures S2 and 2a. To confirm the elemental composition of the exfoliated Bi_2_O_2_Se nanoflakes, encompassing Bi, Se, and O elements, Energy-Dispersive X-ray (EDX) elemental mapping was conducted as shown in Figure 2b–e. The corresponding EDX spectrum (Figure 2b) highlights the elemental composition collected from the selected area, marked with a yellow rectangle, respectively. It is important to note that the carbon peak originated from the carbon substrate or adsorbed species. Interestingly, after the electrochemical reduction, the stoichiometric ratio of elements was changed, revealing an increased atomic percentage of elemental Bi, likely arising from the reduction of the bulk crystal to metallic Bi, while Se stayed in tacked. The elemental mapping for oxygen indicates the presence of adsorbed species throughout the exposed area, with the collected signal showing oxygen as a constituent element within the marked region.

**Figure 3:**
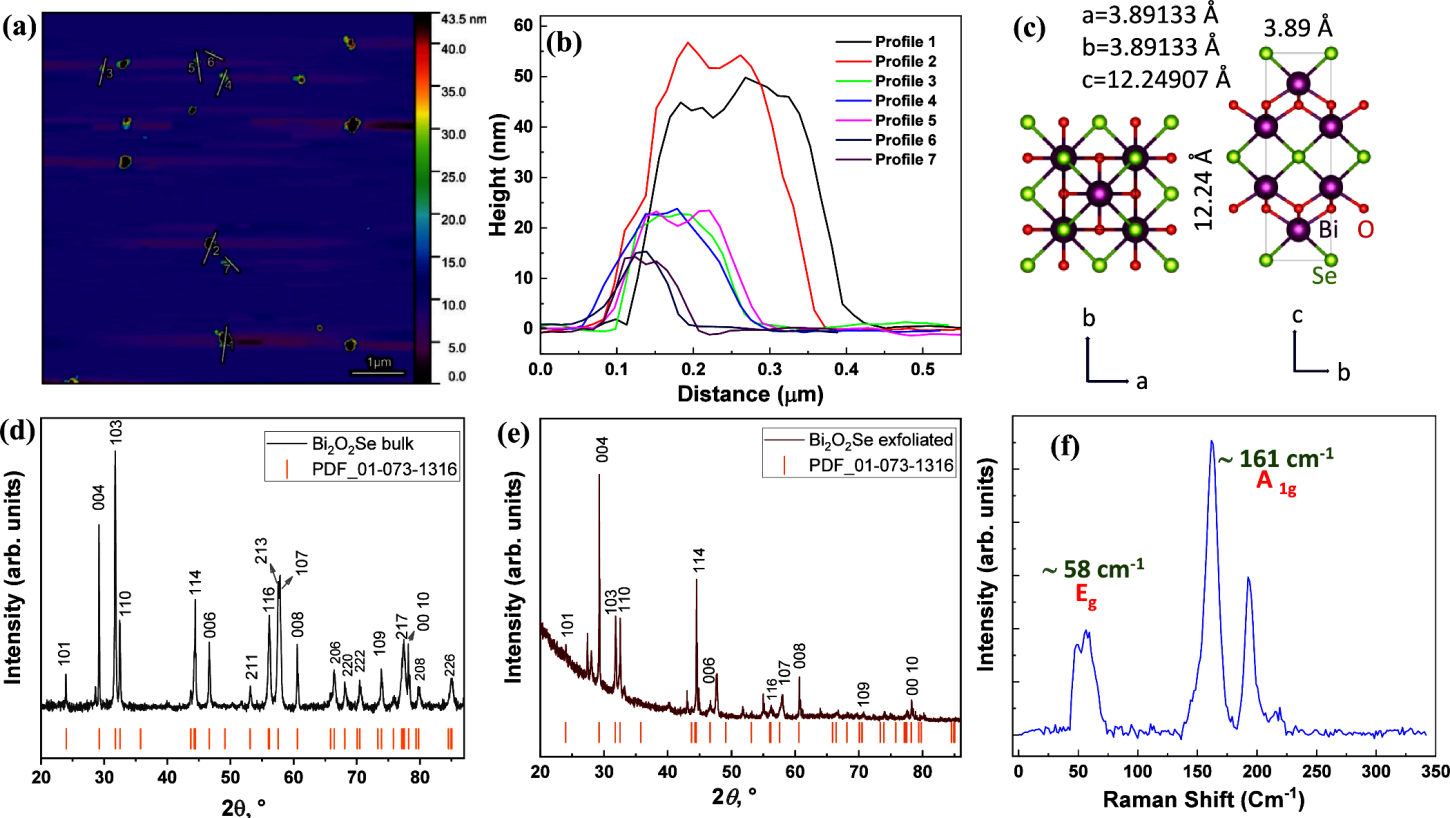
Comprehensive analysis of Bi_2_O_2_Se, (a) Atomic Force Microscopy (AFM) image, revealing the nanoscale morphology. (b) Corresponding size distribution of the particles depicted in Figure (a). (c) Top and side views of the crystal structures. X-ray diffractometer (XRD) measurements of (d) bulk and (e) exfoliated Bi_2_O_2_Se, with marked peaks that correspond to distinct crystallographic planes. (f) Raman spectroscopy measurements of the exfoliated materials, highlighting the associated peaks and their assigned vibrational modes.

The AFM images provide clear confirmation of the exceptional performance achieved through electrochemical exfoliation. This method yielded Bi_2_O_2_Se nanoflakes with thicknesses ranging from approximately 10–80 nm, which falls within the typical range for exfoliated 2D materials, as depicted in Figure 3a. These exfoliated Bi_2_O_2_Se nanoflakes have lateral dimensions between 0.1 and 0.3 μm, ensuring that no significant increase in thickness leading to the formation of multilayered flakes, as evident in the profile shown in Figure 3a. The size distribution of the exfoliated materials is visually represented in Figure 3b. The Bi_2_O_2_Se crystal possesses body-centered tetragonal lattice with conventional values of *a* = *b* = 3.89 Å and *c* = 12.25 Å. Figure 3c shows both top and side views of the crystal structures. The purple, red, and green spheres represent Bi, O, and Se atoms, respectively. Layered structure consists of [Bi_2_O_2_]_*n*_^2*n*+^ and [Se]_*n*_^2*n*–^ layers connected by weak electrostatic forces along the *c* axis. Figure 3d represents X-ray diffractogram of Bi_2_O_2_Se bulk, the pattern confirms a successful synthesis of crystal (PDF 01-073-1316). The tetragonal Bi_2_O_2_Se reveals the reflection of 004 plane at ≈29.12°, suggesting the c lattice parameter of 12.26 Å, then, 101 plane reflection at ≈23.94° which gives the *a* lattice parameter of 3.89 Å. The diffraction pattern of exfoliated Bi_2_O_2_Se shows that material persists in its crystallinity showing well-defined peaks, as shown in Figure 3e. The majority of the peaks occur along the ⟨*k*0*l*⟩ direction.

Raman spectroscopy offers a nondestructive approach for characterizing materials, providing valuable insights into their structural, crystalline, strained, and defective properties by analyzing specific molecular vibration modes. Theoretical calculations using group theory reveal ten vibrational modes for Bi_2_O_2_Se. However, only A_1g_, B_1g_, and E_g_ modes exhibit Raman activity among them.^29^ In exfoliated Bi_2_O_2_Se, two distinct Raman vibration modes manifest at the Γ-point: 161 cm^–1^ for A_1g_ and 57.8 cm^–1^ for E_g_, as depicted in Figure 3f. These modes are consistent with the Raman spectra of the bulk material (Figure S3).^9,29^ The A_1g_ modes correspond to the motion of Bi and O atoms along the crystallographic *Z*-axis, while the E_g_ modes are attributed to the vibration of Bi and O atoms within the *XY*-plane.^30^

**Figure 4:**
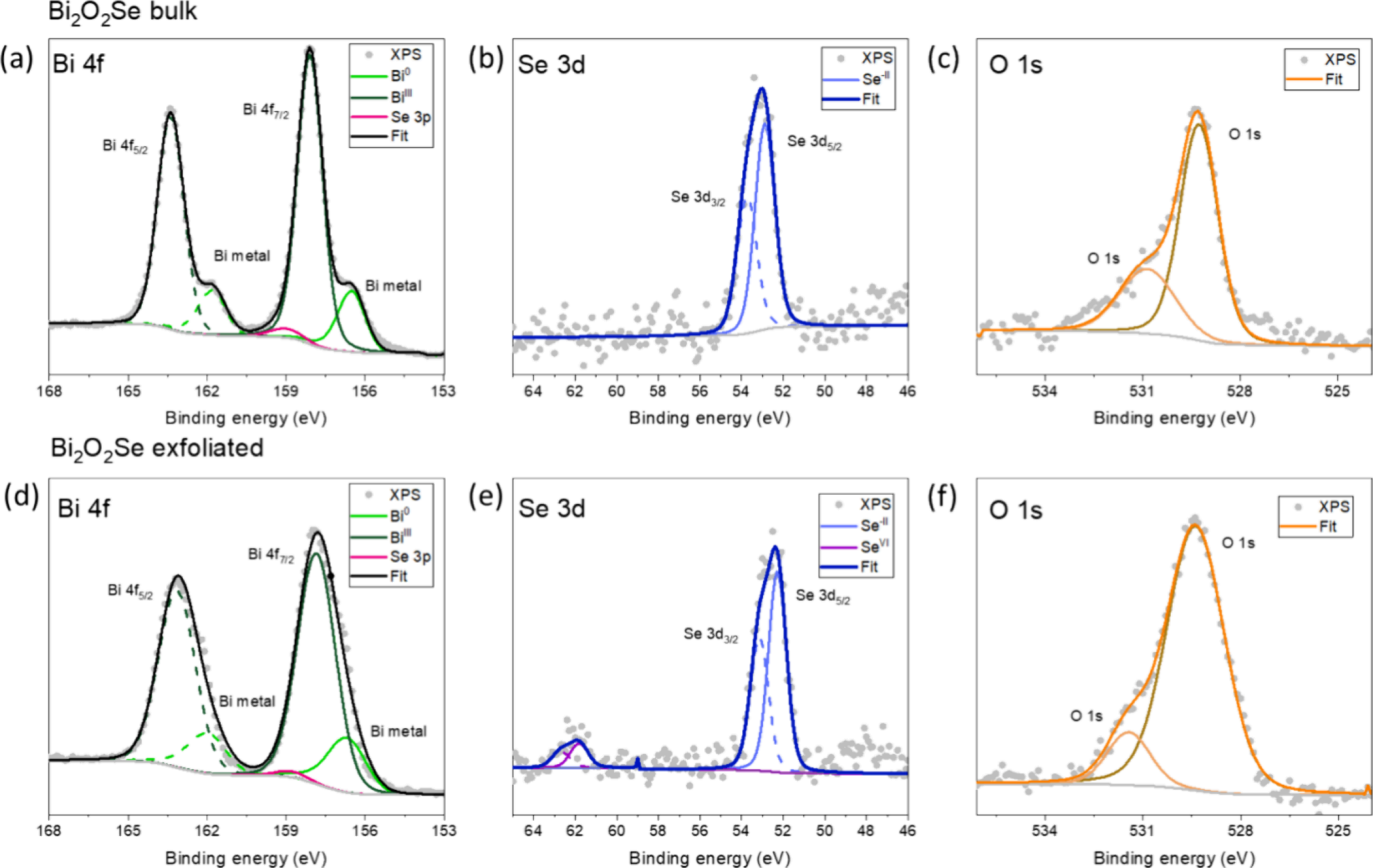
High-resolution X-ray Photoelectron Spectroscopy (XPS) spectra were obtained for both bulk and exfoliated Bi_2_O_2_Se. Figures (a–c) present the XPS spectra of Bi, Se, and O, respectively, for bulk Bi_2_O_2_Se. Similarly, Figures (d–f) show the corresponding XPS spectra for exfoliated Bi_2_O_2_Se.

We analyze the surface composition using wide-scan X-ray photoelectron spectra (XPS). Figure 4 presents detailed high-resolution spectra of Bi, Se, and O for both the bulk material and electrochemically exfoliated Bi_2_O_2_Se. For bulk Bi_2_O_2_Se, the peak at 156.5 eV corresponds to metallic Bi (Figure 4a). In the exfoliated Bi_2_O_2_Se, metallic Bi content increases due to the electrochemical reduction of the material during the exfoliation procedure, while the peak is positioned at 156.7 eV (Figure 4d). Both the bulk and exfoliated materials exhibit a pair of peaks at 52.5 eV corresponding to selenide in Se 3d spectrum (Figure 4b,e). Finally, the O 1s spectra in Figure 4c,f highlight the presence of Oxygen from Bi_2_O_2_Se at 529.3 eV with adventitious oxygen originating from contamination of the samples.

We analyze the surface composition using wide-scan X-ray photoelectron spectra (XPS). Figure 4 presents detailed high-resolution spectra of Bi, Se, and O for both the bulk material and electrochemically exfoliated Bi_2_O_2_Se. In the Bi 4f region, the signal overlaps with Se 3p (Figure 4a,d), and the spin–orbit components are separated by 5.3 eV. For bulk Bi_2_O_2_Se, the peak at 156.5 eV corresponds to metallic Bi (Figure 4a). In the exfoliated Bi_2_O_2_Se, metallic Bi content increases due to the electrochemical reduction of the material during the exfoliation procedure, while the peak is positioned at 156.7 eV (Figure 4d). Both the bulk and exfoliated materials exhibit a pair of peaks at 52.5 eV corresponding to selenide in Se 3d spectrum (Figure 4b,e). In the exfoliated sample, an additional pair of peaks appears at 62.5 eV, indicating the presence of oxidized Se^VI^, likely formed at defects or edges during exfoliation (Figure 4d). Finally, the O 1s spectra in Figure 4c,f show the presence of oxygen from Bi_2_O_2_Se at 529.3 eV, alongside adventitious oxygen due to sample contamination.

**Figure 5:**
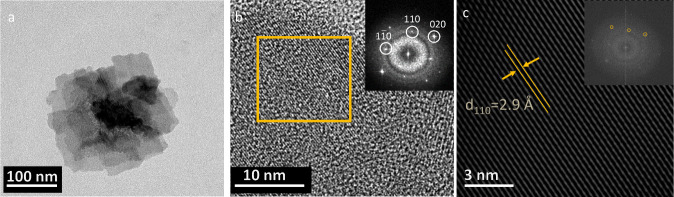
TEM characterization of exfoliated Bi_2_O_2_Se: (a) Low-magnification TEM image, (b) HR-TEM image revealing the Bi_2_O_2_Se lattice structure, and (c) inverse FFT image from the highlighted square region.

The exfoliated Bi_2_O_2_Se was further characterized using TEM. Figure 5a presents a plan-view TEM image, while Figure 5b shows a high-resolution TEM (HR-TEM) image of exfoliated Bi_2_O_2_Se deposited onto a copper support membrane. The HR-TEM image in Figure 5b highlights the high crystallinity of the exfoliated material. The corresponding selected area electron diffraction (SAED) pattern, shown in the inset, clearly demonstrates the tetragonal structure of the sample, with indexed [020], [110], and [1̅1̅0] crystallographic planes. The fast Fourier transform (FFT) pattern of the yellow rectangular region in Figure 5b, displayed in the inset of Figure 5c, further confirms the tetragonal crystal structure of Bi_2_O_2_Se. The inverse FFT (IFFT) image in Figure 5c, derived from the yellow circled reflections in the FFT pattern, verifies the tetragonal symmetry of the Bi/Se atomic columns along the [110] direction.

**Figure 6:**
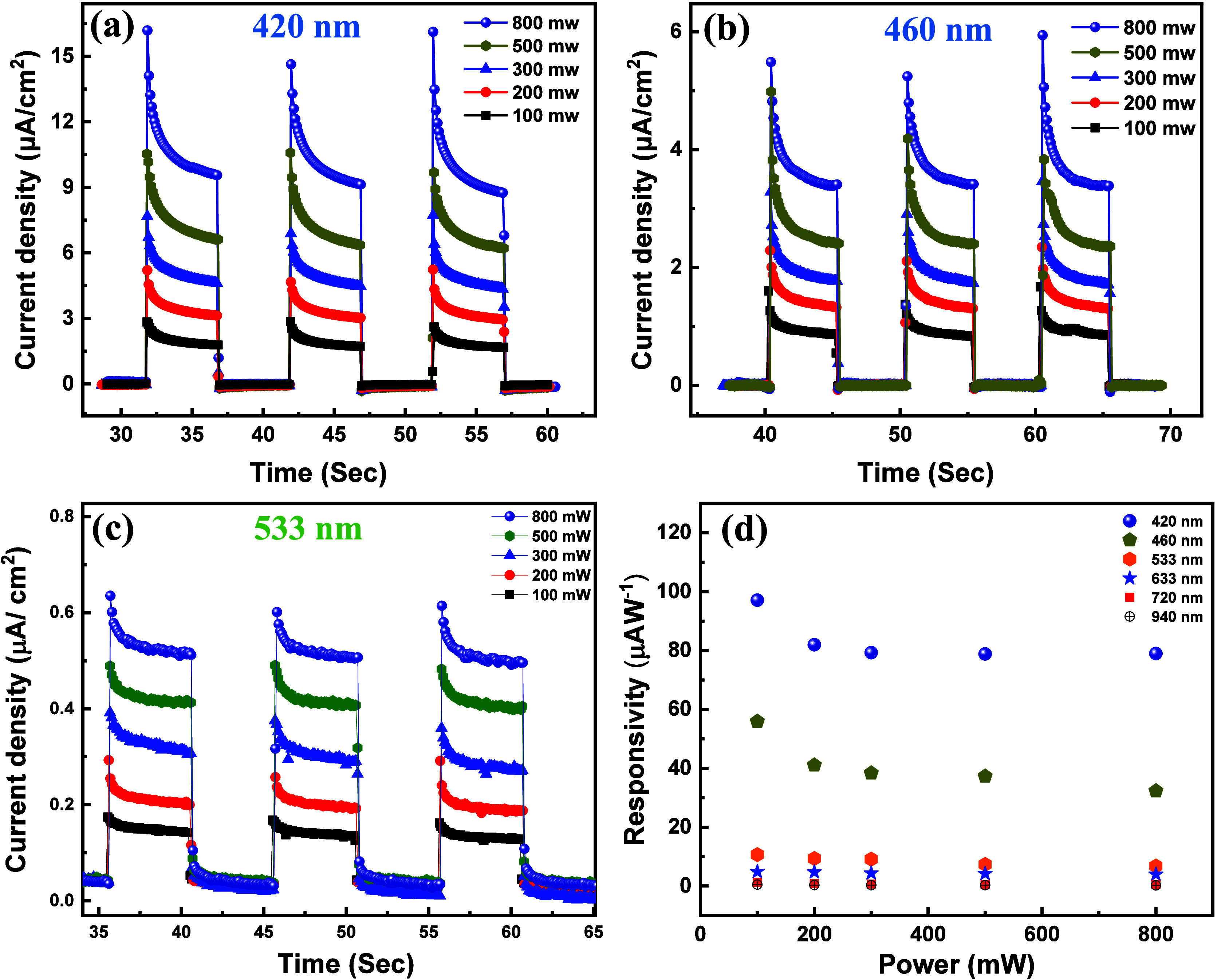
Photocurrent density characteristics of PEC photodetectors employing a few layers of Bi_2_O_2_Se under illumination by LEDs with wavelengths of (a) 420 nm, (b) 460 nm, and (c) 532 nm, all operated at an applied voltage of 0.5 V vs SCE, with varying light intensities. Power of the LEDs was systematically increased from 100 to 800 mW. (d) Power-dependent responsivity of PEC-type photodetectors in a 1 M KOH solution, showcasing their performance across six distinct illumination wavelengths.

We evaluated the photoelectrochemical properties of exfoliated Bi_2_O_2_Se using a three-electrode system. In this setup, the counter electrode was made of platinum, the reference electrode was a saturated calomel electrode (SCE), and the working electrode was an indium tin oxide (ITO) electrode loaded with the exfoliated material. To initiate our investigation, we first studied the current–voltage (I-V) characteristics of the Bi_2_O_2_Se-based PEC photodetector using linear sweep voltammetry (LSV) at a scanning rate of 10 mVs^–1^ under 420 nm LED illumination (Figure S4 a,b). The results showed a clear and consistent increase in current density alongside the applied voltage increase, marked by a distinct on–off response: a rise in current when the light source was on and a corresponding decrease when the light source was off. Notably, it is crucial to highlight that the current experiences exponential growth beyond 0.8 V vs SCE. For precautionary reasons, we have limited our applied voltage to + 0.5 V vs SCE to prevent oxidative conditions that may trigger uncontrolled (photo) electrochemical deterioration of the photoelectrodes. We also measured the power-dependent photoresponse of exfoliated Bi_2_O_2_Se was initially measured in a 1 M KOH solution at an applied voltage of 0.5 V vs SCE under 420 nm LED illumination (Figure 6a). The current density of the PEC photodetector increased from 3 to 16 μA cm^–2^ as the light power was raised from 100 to 800 mW. This rise in current density can be attributed to the enhanced generation of electron–hole pairs as the power increases, resulting in an overall enhancement of the current density. The broadband photoresponse of the photodetector plays a pivotal role in their practical utility, allowing these materials to effectively react to a wide spectrum of light wavelengths. In our experiments, we systematically assessed the photoresponse of exfoliated Bi_2_O_2_Se at various wavelengths, namely 460, 533, 633, 720, and 940 nm, as illustrated in Figures 6b,c and S5a–c, respectively.

The performance of PEC photodetectors has undergone significant advancements, achieved through the exploration of various key parameters. Among these critical factors, responsivity (*R*_ph_) emerges as a pivotal metric. Responsivity, denoted as *R*_ph_, is precisely defined as the change in current density divided by the power of the incident irradiation signal. This essential parameter can be quantified using the following equation:
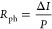
1where Δ*I* represents the increment in current density relative to the dark current, and P signifies the intensity of irradiation power per unit area.^28^ The Bi_2_O_2_Se photodetector demonstrates a notable responsivity of approximately 97.1 μAW^–1^ under 420 nm illumination (Figure 6d). The assessed responsivity (*R*_ph_) of the Bi_2_O_2_Se photodetector device, analyzed across various illumination wavelengths and power levels, is also depicted in Figure 6d. This graphical representation clearly indicates that the photodetector achieves its highest responsivity with blue LED illumination, gradually decreasing as the illumination shifts from green to infrared. A comparison between the calculated responsivity and published results is presented in Table S1, highlighting that the device’s responsivity is either superior to or comparable with previous reports.^26,31−43^ A comprehensive comparison of Bi_2_O_2_Se-based photodetectors, including their response times, with previously published results and other types of photodetectors is also provided in Tables S2 and S3.

Another crucial parameter to consider, alongside responsivity, is Specific Detectivity (D*), which can be calculated using the following formula:^44,45^
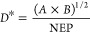
2

Here, the symbols *A*, *B*, and NEP correspond to the active area of the photodetector device, the measured bandwidth, and the noise equivalent power, respectively. Notably, NEP represents the signal power at which a signal-to-noise ratio (SNR) of 1 is achieved. This value signifies the minimum optical power required for the photodetector to differentiate signal from noise effectively.

Alternatively, the Noise-Equivalent Power (NEP) can be expressed using the following formula^44,46^
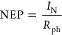
3

Here, *R*_ph_ represents the photoresponsivity of the photodetector device, while *I*_N_ corresponds to the noise current. Additionally, the noise can be closely approximated by the dark current (*I*_D_) of the photodetector device and can be described by the equation:
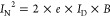
4

**Figure 7:**
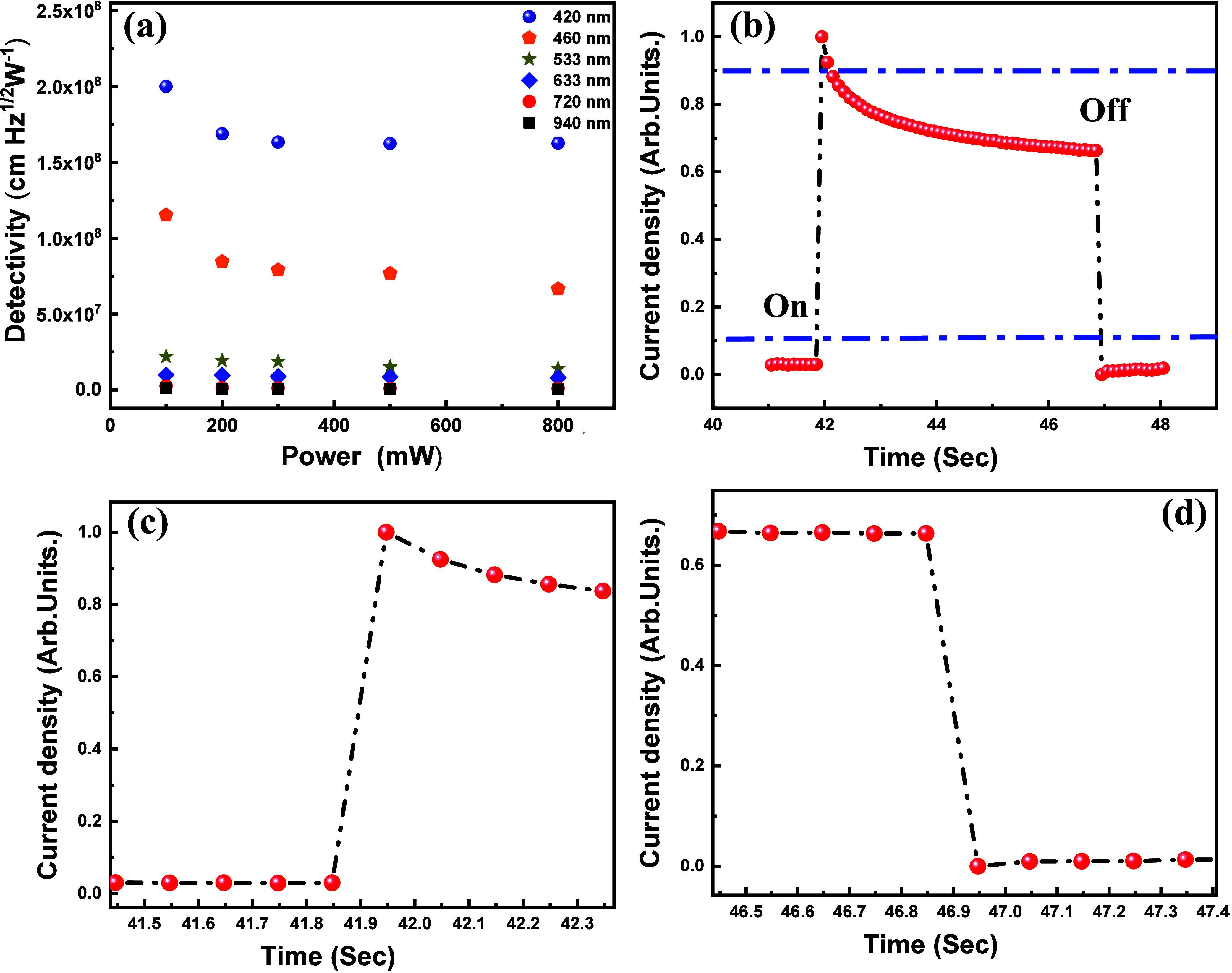
(a) Specific detectivity of PEC-type Bi_2_O_2_Se-based photodetectors in a 1 M KOH solution under six distinct illumination wavelengths. Device’s response time is detailed in subfigures (b–d). In (b), the normalized current density changes from 10 to 90%, while (c,d) provide a closer look at the rise and fall times of the device, respectively.

In this equation, ‘e’ denotes the electronic charge.^45,47,48^ By combining eqs 2 and 3, we determined that the specific detectivity of our photodetector devices is approximately 2 × 10^8^ cmHz^1/2^ W^–1^ (Figure 7a). This relationship between specific detectivity, power, and various illumination wavelengths is visually depicted in Figure 7a. We have also evaluated the external quantum efficiency (η) of the Bi_2_O_2_Se photodetector, as described in the Supporting Information. The results show a maximum η of approximately 0.03% under 420 nm illumination (Figure S6). The performance of photodetector devices in practical applications is heavily reliant on their response time, a critical parameter. In our study, we employed various current density levels (ranging from 10 to 90%) following established convenience methods. Subsequently, we calculated the device’s response time under 420 nm light (500 mW) in a 1 M KOH electrolyte. The results, illustrated in Figure 7b, revealed remarkably fast rise, and fall times of 82 ms (Figure 7c) and 86 ms (Figure 7d), respectively. This phenomenon can be attributed to the solution’s preferential and swift electron transfer processes. The photoresponse characteristics of Exfoliated Bi_2_O_2_Se were further investigated in a 0.5 M Na_2_SO_4_ solution, applying a voltage of 0.5 V vs SCE under 420 nm LED illumination (see Figure S7a). Further photoresponse experiments were conducted with varying illumination wavelengths, as illustrated in Figure S7b (460 nm) and S7c (533 nm). Consistent with the KOH solution results, a strong response was observed at 420 nm, followed by a gradual decline in response at 460 and 533 nm LED illumination, as depicted in Figure S7b,c, respectively. The calculated photoresponsivity and specific detectivity of PEC-type Bi_2_O_2_Se-based photodetectors in a 1 M Na_2_SO_4_ solution, showcasing their performance across five distinct illumination wavelengths, are presented in Figure S8a,b, respectively. Under these conditions, the photodetector demonstrated a responsivity of approximately 2.5 μAW^–1^ and specific detectivity of 4.9 × 10^6^ cm Hz ^1/2^ W^–1^ at an applied voltage of −0.5 V vs SCE during 420 nm LED illumination. Notably, the exfoliated sample exhibited an unstable photoresponse in a 0.5 M H_2_SO_4_ solution. Although these values are slightly lower compared to the results obtained in the KOH solution, but still remain comparable to published results (refer to Supporting Information Table S1).

**Figure 8:**
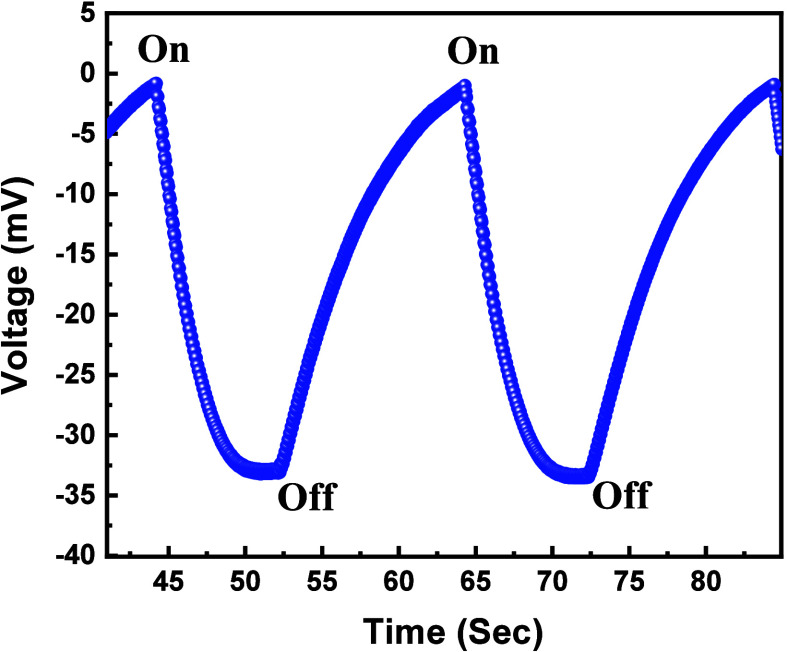
Open-circuit potential (OCP) measurements of a photodetector device based on a photoelectrochemical (PEC) setup in a 1 M KOH solution over time. OCP measurement was conducted under an 800-mW illumination, ensuring the complete resolution of band bending.

Here, we present a self-powered photodetector that incorporates Bi_2_O_2_Se within a photoelectrochemical (PEC) configuration. This phenomenon involves the absorption of light by the Bi_2_O_2_Se photocathode, leading to the creation of electron–hole pairs. Subsequently, these charges are collected at the electrodes, generating a current that correlates with the intensity of the incident light. The self-powered functionality was initially assessed by measuring the photoresponse at 0 V vs SCE under two distinct light illuminations: 420 and 460 nm, as illustrated in Figure S9a,b, respectively. We also assessed the open-circuit potential of the photodetector, which aligns with the potential accumulation caused by the interaction between the materials in the depleted electrolyte. These interactions have been extensively discussed in our previous research papers.^27,48^ In essence, the energy levels of the material and the solution’s Fermi level were mismatched prior to immersion. Upon immersing the semiconductor in the solution, band bending ensues as charge transfer takes place to equate the Fermi level of the semiconductor with that of the solution, leading to charge separation within the semiconductor and subsequent potential accumulation.^48−50^ The band bending leads to the accumulation of positive charge at the surface of the semiconductor, as indicated by the negative Hall coefficient, which confirms that Bi_2_O_2_Se exhibits *n*-type polarity (see Supporting Information). Simultaneously, electrons migrate in the opposite direction, driven by the band bending, leading to the establishment of a voltage within the semiconductor. When light is incident on the semiconductor, a reverse band bending occurs, gradually restoring the band bending to its original position. The built in voltage can be quantified experimental using the open-circuit potential, as shown in Figure 8. This voltage was observed to be approximately 30 mV in a 1 M KOH solution when exposed to 420 nm LED light (800 mW), providing solid evidence for the photodetector’s self-powering capability.^27^

The stability of Bi_2_O_2_Se is a key attribute for its potential application as a photocathode in self-powered photodetectors based on PEC principles. Remarkably, Bi_2_O_2_Se exhibits exceptional stability when subjected to electrochemical conditions and remains resilient even after prolonged exposure to light. This robustness is further illustrated in Figure S10a–c, where the photocurrent demonstrates remarkable stability for over 2000 s. The slight reduction in current density observed during measurements is likely due to material loss in the solution. This inherent stability proves to be a pivotal factor to consider when contemplating the extended operational lifespan of PEC self-powered photodetectors. Furthermore, the combination of Bi_2_O_2_Se-based PECs’ elevated photocurrents and exceptional stability, along with their self-powering capability, positions these devices as a highly promising solution across a diverse spectrum of applications.

## 4 Conclusions

We successfully synthesized Bi_2_O_2_Se and exfoliated it into few layers using electrochemical exfoliation. The resulting exfoliated materials were employed as efficient photocathodes in self-powered photoelectrochemical photodetectors, achieving remarkable photoresponsivity and specific detectivity values of 97.1 μAW^–1^ and 2 × 10^8^ cm Hz^1/2^ W^–1^, respectively, at an applied voltage of 0.5 V relative to SCE under 420 nm LED illumination. Moreover, this photodetector demonstrated an ultrafast response with rapid rise (82 ms) and fall times (86 ms), attributed to the rapid electron transfer processes in the solution, coupled with exceptional stability. These qualities render it highly suitable for practical applications. Our results highlight the potential advantages of PEC-based photodetectors, underscoring their promise as candidates for high-performance optoelectronic applications.

## Data Availability

The datasets generated during and/or analyzed during the study are accessible via the Zenodo repository: https://zenodo.org/records/13956734, https://doi.org/10.5281/zenodo.13827444

## Supporting Information Available

Electrochemical exfoliation, top-view SEM image, IV characteristic curve, photocurrent density with different wavelengths, responsivity and detectivity of PEC-type photodetectors, comparison table, photoresponse of photodetector at 0 V vs SCE, open circuit potential, and stability of photodetector (PDF)
