# Supplementary material for: Next-Generation Self-Powered Photodetectors using 2D Bismuth Oxide Selenide Crystals
Source: ACS Appl Nano Mater. 2024 Oct 23;7(21):24377–87. doi: 10.1021/acsanm.4c03594 (PMC11555640; doi:10.1021/acsanm.4c03594)
Supplement: Supplementary file 1 — an4c03594_si_001.pdf [file an4c03594_si_001.pdf]

Supporting Information

Next-Generation Self-Powered Photodetectors using 2D Bismuth Oxide Selenide Crystals

*Pradip Kumar Roy, *[a] Kseniia Mosina, [a] Sofia Hengtakaeh [a] Kalyan Jyoti Sarkar, [a] Vlastimil Mazánek, [a] Jan Luxa,[a] and Zdenek Sofer* [a]*

[a] Department of Inorganic Chemistry, University of Chemistry and Technology Prague, Technická 5, 166 28 Prague 6, Czech Republic

E-mail: royp@vscht.cz, zdenek.sofer@vscht.cz

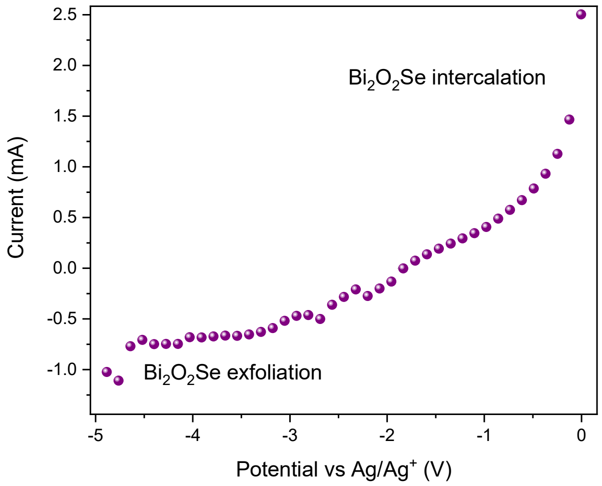

Figure S1. The electrochemical exfoliation process with the application of a potential.

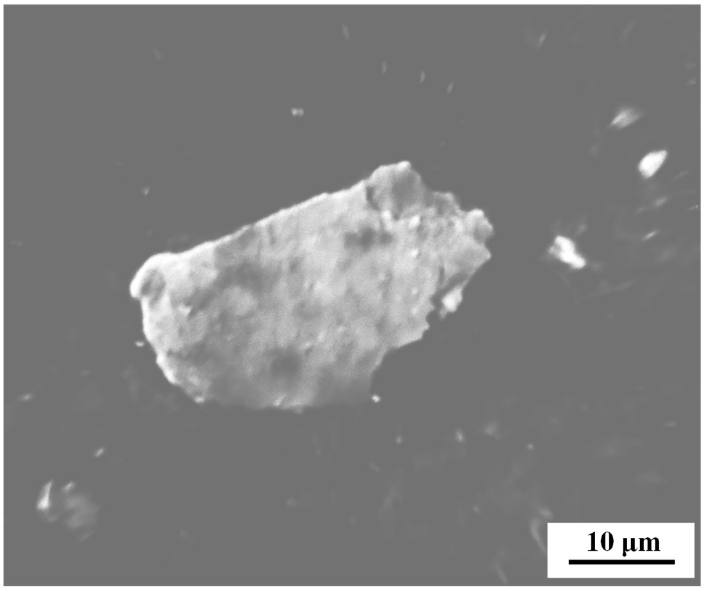

Figure S2. Top-view scanning electron microscope (SEM) image reveals the distinctive flake-like structure of exfoliated Bi_2O_2Se.

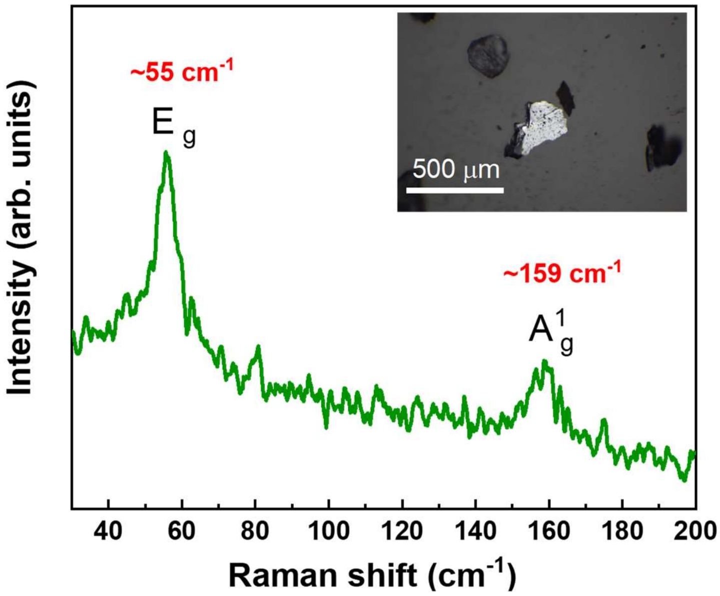

Figure S3. Raman spectroscopy measurements of the bulk materials, highlighting the associated peaks and their assigned vibrational modes.[S1]

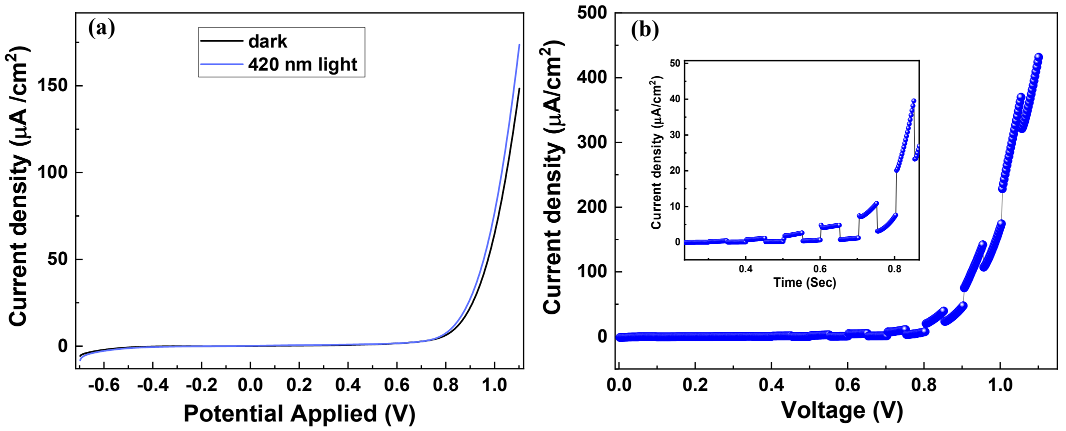

Figure S4. The current-voltage (IV) characteristics of the Bi_2O_2Se-based PEC photodetector. (a) IV characteristics with dark and light source (b) on-off response with chop on and off light sources were obtained using a linear sweep voltammetry (LSV) approach at a scanning speed of 10 mV s^{-1}, and the system was illuminated with a 420 nm LED. Notably, the results reveal a distinct on-off response.

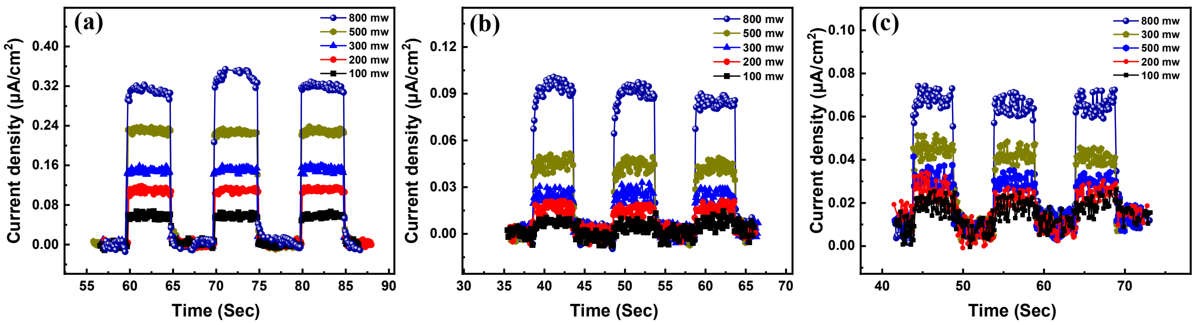

Figure S5. The photocurrent density characteristics of PEC photodetectors employing a few layers of Bi_2O_2Se under illumination by LEDs with wavelengths of (a) 633 nm, (b) 720 nm, and (c) 940 nm, all operated at an applied voltage of 0.5 V relative to SCE in a 1 M KOH solution. The power of the LEDs was systematically increased from 100 to 800 mW.

Table S1. A comprehensive comparison of the performance of a photodetector based on Bi_2O_2Se with previously published results.

Materials	Device configuration	Measurement conditions		Responsivity (mA W^{-1})	Wavelength (nm)	Reference
		Electrode	Applied Potential			
GaSe nanosheets	PEC-type	0.5 M H_2SO_4	-0.3 V *vs.* RHE	160	455	*23*
				19.5	455	
GeSe nanosheets	PEC-type	0.1 M KOH	0.3 V *vs.* SCE	0.044	Simulated sunlight	31
				0.076	Simulated sunlight	
Rh–Cr2O3 modified p-AlGaN nanowires.	PEC-type	0.5 M H_2SO_4	0 V *vs.* Ag/AgCl	183	255 nm	*32*
lignin-derived GQDs	PEC-type	0.01 M KCl	0.6 V *vs.* SCE	0.003	Simulated sunlight	*33*
SnSe2/SnSe hetero-structures	PEC-type	0.5 M Na2SO4	0.4 V *vs.* SCE	0.523	475	*34*
InSe nanosheets	PEC-type	0.3 M KOH	0 V *vs.* SCE	10.14	365	*35*
GeSe nanosheets	PEC-type	0.1 M KOH	0.3 V *vs.* RHE	0.044	Simulated sunlight	*36*
In_2O_3 microrods	PEC-type	0.5 M H_2SO_4 1 M KOH	0.6 V *vs.* Ag/AgCl	21.19	365	*37*
InSe nanosheets	PEC-type	0.2 M KOH	1 V *vs.* SCE	3.3×10^{-3}	Simulated sunlight	*38*
				4.9×10^{-3}	Simulated sunlight	
Black phosphorous nanosheets	PEC-type	0.1 M KOH	0 V *vs.* SCE	1.9×10^{-3}	Simulated sunlight	*39*
				2.2×10^{-3}	Simulated sunlight	
Perovskite ($CH_3NH_3PbI_3$)	Metal-semiconductor-metal	0.1 M KOH	5 V	4.4	633	*40*
SnS	PEC-type	0.1 Na_2SO_4	0.6 V	0.018	365	*41*
PBDTT-ffQx/PCBM bulk heterojunction	Metal-semiconductor-metal	-	10 V	1.15×10^3	365	*42*
SnS/RGO hybrid nanosheets	FET	-	V_{DS} =5V, V_g = 0 V	180	visible light	*43*
**Bi_2O_2Se**	**PEC-type**	**1 M KOH**	**0.5 V *vs.* SCE**	**0.097**	**420 nm**	**This Work**

Table S2. A comprehensive comparison of response time of a photodetector based on Bi_2O_2Se with previously published results.

Materials	Device configuration	Measurement conditions		Responsivity (mA W^{-1})	Response time	Reference
		Electrode	Wavelength (nm)			
Few-layers BP	PEC-type	0.1 M KOH	Simulated sunlight	2.65	0.5 Sec	S2
2D Te nanosheets	PEC-type	0.1 M KOH	Simulated sunlight	43×10^{-6}	54.5 ms	S3
2D Bi nanosheets	PEC-type	1 M NaOH	Simulated sunlight	0.152	0.3 sec	S4
Few-layers Bi2S3	PEC-type	0.1 M KOH	365 nm	8.9	0.1 sec	S5
Few-layers SnS	PEC-type	0.1 M Na2SO4	365 nm	5.2	0.3 sec	S6
lignin-derived GQDs	PEC-type	0.01 M KCl	Simulated sunlight	0.003	1.5 sec	S7
SnSe2/SnSe hetero-structures	PEC-type	0.5 M Na2SO4	475 nm	0.523	13 ms	S8
In_2O_3 Nano cube	PEC-type	1 M KOH	365	44.43	20 ms	S9
InSe nanosheets	PEC-type	0.3 M KOH	365	10.14	2 ms	S10
**Bi_2O_2Se**	**PEC-type**	**1 M KOH**	**420 nm**	**0.097**	**82 ms**	**This Work**

Table S3. A comprehensive comparison of our PEC photodetector with others types of photodetectors.

Materials	Device configuration	Measurement conditions	Responsivity (A W^{-1})	Reference
		Applied Potential		
$NiTe_2/WS_2$ heterostructures	FET	V_{ds}= 1 V, V_g= -40-80 V	0.3	S11
MoS_2 nanosheets	Schottky-contact	V_{ds}= 2 V, V_g= -50 V	15	S12
BP/ $MAPbI_{3-x}Cl_x$	Schottky-contact	V_{ds}= -2 V, V_g= 30 V	10^8	S13
Few-layer BP flakes	Schottky-contact	V_{ds}= -1 V, V_g= 10 V	3.5×10^{-3}	S14
Single-Layer MoS_2	FET	V_{ds}= 1 V, V_g= 0 V	420×10^{-6}	S15
Vertically Aligned SnS2 and RGO	*p-n* junction	V_{ds}= 2 V, V_g= 5 V	1.3	S16
Ti_3C_2Tx MXene/Si	Schottky-contact	V_{ds}= -2 V, V_g= 0 V	0.3	S17
Layered InSe	FET	V_{ds}= 1 V, V_g= 0 V	27	S18
SnS_2	Metal-semiconductor-metal	V_{ds}= 10 V, V_g= 0 V	2	S19
Bi_2O_2Se	PEC-type	0. 5 V *vs.* SCE	97×10^{-6}	This Work

External Quantum Efficiency (EQE, η)

External quantum efficiency (η) is defined as the ratio of electron-hole pairs generated to the number of incident photons. The equation for calculating the quantum efficiency is shown below: [S20]

$$\mathrm{EQE} = \eta = \frac{hc\,R}{q\lambda} \qquad \text{................. Equation S1}$$

where:

- λ is the wavelength of the incident light,
- c is speed of light,
- R is the responsivity of the device,
- h is Planck's constant,
- q is the elementary charge.

The calculated η is plotted in Figure S as a function of different wavelengths.

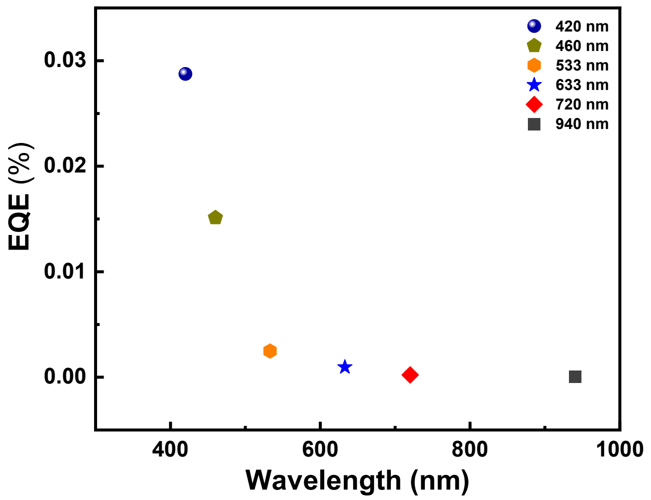

Figure S6. External quantum efficiency (EQE) of the devices with different wavelength.

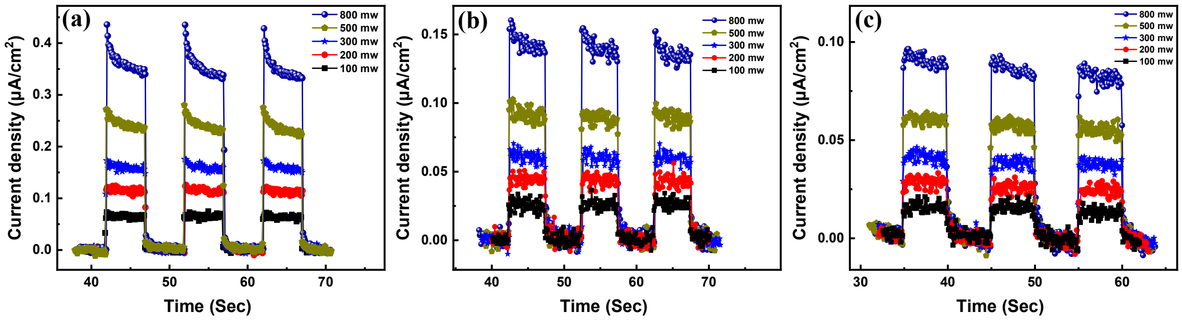

Figure S7. The photocurrent density characteristics of PEC photodetectors employing a few layers of Bi_2O_2Se under illumination by LEDs with wavelengths of (a) 420 nm, (b) 460 nm, and (c) 533 nm, all operated at an applied voltage of 0.5 V relative to SCE in a 1 M Na_2SO_4 solution. The power of the LEDs was systematically increased from 100 to 800 mW.

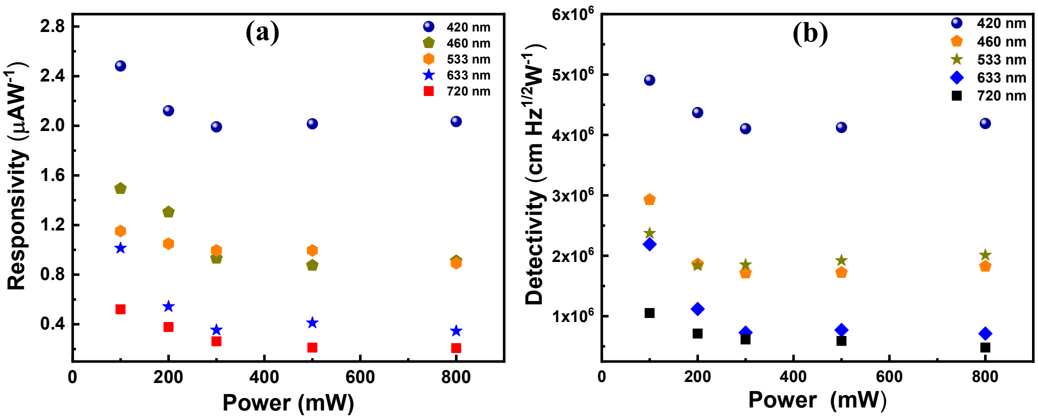

Figure S8. Depicts the power-dependent (a) Responsivity and (b) The specific detectivity of PEC-type Bi_2O_2Se -based photodetectors in a 1 M Na_2SO_4 solution, showcasing their performance across five distinct illumination wavelengths.

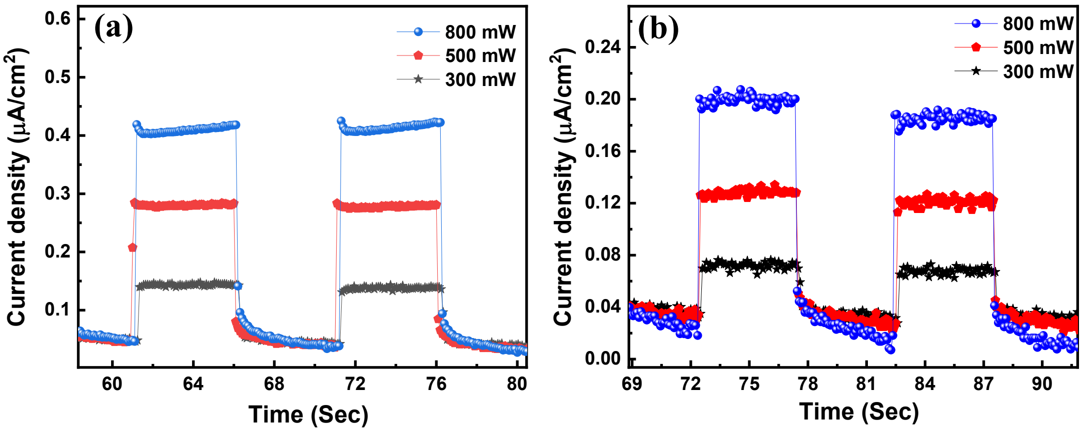

Figure S9. The photocurrent density characteristics of PEC photodetectors under two distinct light illuminations: (a) 420 nm and (b) 460 nm. These photodetectors were operated at an applied voltage of 0 V relative to SCE while varying the power.

Hall measurements

In order to confirm the polarity of the Bi_2O_2Se we further conducted Hall measurements using DX-100 Hall effect system in Van der Pauw geometry on bulk crystals in room temperature. Rectangular shaped crystal was chosen and silver paste was deposited on the corners of the sample as a contact electrode, the thickness of the sample was measured as 2 mm. In the measurement magnetic field was swept from -500 mT to +500 mT and current was applied 1mA, and every time the Hall voltage and Hall coefficient found to be negative which signifies that Bi_2O_2Se has n type polarity. The bulk carrier concentration was calculated as 4.09×10^{14} cm^{-3}.

Thickness d (cm)	Magnetic Field (mT)	Temperature (K)	Current (mA)	Hall Voltage (mV)	Hall coefficient (cm^3/C)	Bulk carrier concentration ($1/cm^3$)
0.2	500	300.05	1	-3.822981	-15291.92	4.09E+14

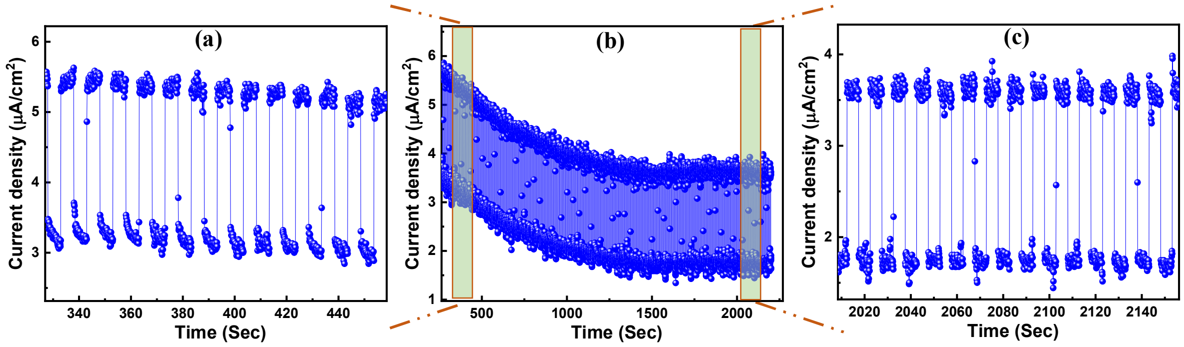

Figure S10. The electrochemical stability of exfoliated Bi_2O_2Se in a 1 M KOH solution. (b) A linear sweep voltammetry analysis of the on/off photocurrent response under a 0.5 V bias (vs. SCE) while subjecting the sample to 500 mW illumination from a 420 nm LED. Subsequently, (a) and (c) provide enlarged on/off cycles extracted from the data in Figure (b), showcasing the photocurrent responses during the initial and final portions of the measurement.

Reference for supporting information

S1. Kim, U. J.; Nam, S. H.; Seo, J.; Yang, M.; Fu, Q.; Liu, Z.; Son, H.; Lee, M.; Hahm, M. G., Visualizing Line Defects in non-van der Waals Bi_2O_2Se Using Raman Spectroscopy. *ACS Nano* **2022,** *16*, 3637-3646.

S2. Ren, X.; Li, Z.; Huang, Z.; Sang, D.; Qiao, H.; Qi, X.; Li, J.; Zhong, J.; Zhang, H., Environmentally Robust Black Phosphorus Nanosheets in Solution: Application for Self-Powered Photodetector. *Adv. Funct. Mater.* **2017,** *27* , 1606834.

S3. Xie, Z.; Xing, C.; Huang, W.; Fan, T.; Li, Z.; Zhao, J.; Xiang, Y.; Guo, Z.; Li, J.; Yang, Z.; Dong, B.; Qu, J.; Fan, D.; Zhang, H., Ultrathin 2D Nonlayered Tellurium Nanosheets: Facile Liquid-Phase Exfoliation, Characterization, and Photoresponse with High Performance and Enhanced Stability. *Adv. Funct. Mater.* **2018,** *28*, 1705833.

S4. Huang, H.; Ren, X.; Li, Z.; Wang, H.; Huang, Z.; Qiao, H.; Tang, P.; Zhao, J.; Liang, W.; Ge, Y.; Liu, J.; Li, J.; Qi, X.; Zhang, H., Two-Dimensional Bismuth Nanosheets as Prospective Photo-Detector with Tunable Optoelectronic Performance. *Nanotechnol.* **2018,** *29*, 235201.

S5. Huang, W.; Xing, C.; Wang, Y.; Li, Z.; Wu, L.; Ma, D.; Dai, X.; Xiang, Y.; Li, J.; Fan, D.; Zhang, H., Facile Fabrication and Characterization of Two-Dimensional Bismuth(iii) Sulfide Nanosheets for High-Performance Photodetector Applications under Ambient Conditions. *Nanoscale* **2018,** *10* , 2404-2412.

S6. Huang, W.; Xie, Z.; Fan, T.; Li, J.; Wang, Y.; Wu, L.; Ma, D.; Li, Z.; Ge, Y.; Huang, Z. N.; Dai, X.; Xiang, Y.; Li, J.; Zhu, X.; Zhang, H., Black-Phosphorus-Analogue Tin Monosulfide: an Emerging Optoelectronic Two-Dimensional Material for High-Performance Photodetection with Improved Stability under Ambient/Harsh Conditions. *J. Mater. Chem. C* **2018,** *6*, 9582-9593.

S7. Wang, R.; Su, W.; Zhang, S.; Jin, L.; Zhang, J.; Bian, H.; Zhang, Y., Application of Lignin-Derived Graphene Quantum Dots in Visible Light-Driven Photoelectrochemical Photodetector. *Adv. Opt. Mater.* **2023,** *11* , 2202944.

S8. Lu, C.; Dong, W.; Zou, Y.; Wang, Z.; Tan, J.; Bai, X.; Ma, N.; Ge, Y.; Zhao, Q.; Xu, X., Direct Z-Scheme $SnSe_2$/SnSe Heterostructure Passivated by Al_2O_3 for Highly Stable and Sensitive Photoelectrochemical Photodetectors. *ACS Appl. Mater. Interfaces.* **2023,** *15*, 6156-6168.

S9. Zhang, N.; Cui, M.; Zhou, J.; Shao, Z.; Gao, X.; Liu, J.; Sun, R.; Zhang, Y.; Li, W.; Li, X.; Yao, J.; Gao, F.; Feng, W., High-Performance Self-Powered Photoelectrochemical Ultraviolet Photodetectors Based on an In_2O_3 Nanocube Film. *ACS Appl. Mater. Interfaces.* **2024,** *16* , 19167-19174.

S10. Yang, X.; Liu, X.; Qu, L.; Gao, F.; Xu, Y.; Cui, M.; Yu, H.; Wang, Y.; Hu, P.; Feng, W., Boosting Photoresponse of Self-Powered InSe-Based Photoelectrochemical Photodetectors via Suppression of Interface Doping. *ACS Nano* **2022,** *16* , 8440-8448.

S11. Qi, Z.; Zhai, X.; Jiang, X.; Xu, X.; Fan, C.; Shen, L.; Xiao, Q.; Jiang, S.; Deng, Q.; Liu, H.; Jing, F.; Zhang, Q., Epitaxy of $NiTe_2$ on WS_2 for the p-Type Schottky Contact and Increased Photoresponse. *ACS Appl. Mater. Interfaces.* **2022,** *14*, 31121-31130.

S12. Dai, M.; Wu, Q.; Wang, C.; Liu, X.; Zhang, X.; Cai, Z.; Lin, L.; Gu, X.; Ostrikov, K.; Nan, H.; Xiao, S., High Performance Self-Driven Photodetectors Based on MoS_2 Schottky Barrier Diode. *Adv. Opt. Mater.* **2024,** *12*, 2301900.

S13. Zou, X.; Li, Y.; Tang, G.; You, P.; Yan, F., Schottky Barrier-Controlled Black Phosphorus/Perovskite Phototransistors with Ultrahigh Sensitivity and Fast Response. *Small* **2019,** *15*, 1901004.

S14. Miao, J.; Zhang, S.; Cai, L.; Wang, C., Black Phosphorus Schottky Diodes: Channel Length Scaling and Application as Photodetectors. *Adv. Elec. Mater.* **2016,** *2*, 1500346.

S15. Yin, Z.; Li, H.; Li, H.; Jiang, L.; Shi, Y.; Sun, Y.; Lu, G.; Zhang, Q.; Chen, X.; Zhang, H., Single-Layer MoS_2 Phototransistors. *ACS Nano* **2012,** *6*, 74-80.

S16. Das, C.; Kumar, S.; Dambhare, N. V.; Kumar, M.; Rath, A. K.; Sahu, S., High-Performance Photodetector from p-n Junction of Vertically Aligned SnS_2 and Reduced Graphene Oxide. *ACS Appl. Electron. Mater.* **2024**, *6,* 6965–6973

S17. Song, L.; Xu, E.; Yu, Y.; Jie, J.; Xia, Y.; Chen, S.; Jiang, Y.; Xu, G.; Li, D.; Jie, J., High-Barrier-Height Ti_3C_2T/Si Microstructure Schottky Junction-Based Self-Powered Photodetectors for Photoplethysmographic Monitoring. *Adv. Mater. Technol.* **2022,** *7* , 2200555.

S18. Yang, Z.; Jie, W.; Mak, C.-H.; Lin, S.; Lin, H.; Yang, X.; Yan, F.; Lau, S. P.; Hao, J., Wafer-Scale Synthesis of High-Quality Semiconducting Two-Dimensional Layered InSe with Broadband Photoresponse. *ACS Nano* **2017,** *11*, 4225-4236.

S19. Xia, J.; Zhu, D.; Wang, L.; Huang, B.; Huang, X.; Meng, X.-M., Large-Scale Growth of Two-Dimensional SnS_2 Crystals Driven by Screw Dislocations and Application to Photodetectors. *Adv. Funct. Mater.* **2015,** *25*, 4255-4261.

S20. Dai, M.; Zhang, X.; Wang, Q. J., 2D Materials for Photothermoelectric Detectors: Mechanisms, Materials, and Devices. *Adv. Funct. Mater.* **2024,** *34*, 2312872.
